# Vertical transmission of SARS-CoV-2 delta-variant in a preterm infant

**DOI:** 10.1186/s12879-024-09420-y

**Published:** 2024-05-28

**Authors:** Muhammad T.K. Zia, Kishan Kumar, Edmund La Gamma, Fauzia Shakeel, Iman Hanna, Xinhua Lin, Nazeeh Hanna

**Affiliations:** 1 Department of Pediatrics, Maria Fareri Children’s Hospital at Westchester Medical Center, New York Medical College, Valhalla, NY USA; 2 Department of Pediatrics, New York-Presbyterian/Hudson Valley Hospital, Cortlandt Manor, NY USA; 3 Division of Neonatology, Department of Pediatrics, NYU Langone Hospital—Long Island, New York University Grossman Long Island School of Medicine, 259 First Street, Mineola, NY 11501 USA; 4 Department of Pathology, NYU Langone Hospital—Long Island, New York University Grossman Long Island School of Medicine, Mineola, NY USA; 5 Women and Children’s Research Laboratory, NYU Langone Hospital—Long Island New York University Grossman Long Island School of Medicine, Mineola, NY USA; 6 Department of Biochemistry & Molecular Biology, New York Medical College, Valhalla, USA

**Keywords:** SARS-CoV-2, COVID-19, Vertical transmission, Preterm infant, Delta variant

## Abstract

**Background:**

As SARS-CoV-2 continues to be relevant and cause illnesses, the effect of emerging virus variants on perinatal health remains to be elucidated. It was demonstrated that vertical transmission of SARS-CoV-2 is a relatively rare event in the original SARS-CoV-2 strain. However, very few reports describe vertical transmission related to the delta-variant.

**Case presentation:**

We report a case of a preterm male neonate born to a mother with positive SARS-CoV-2 and mild respiratory complications. The neonate was born by cesarean section due to fetal distress. The rupture of the amniotic membrane was at delivery. The neonate had expected prematurity-related complications. His nasopharyngeal swabs for RT-PCR were positive from birth till three weeks of age. RT-ddPCR of the Placenta showed a high load of the SARS-CoV-2 virus with subgenomic viral RNA. RNAscope technique demonstrated both the positive strand of the S gene and the orf1ab negative strand. Detection of subgenomic RNA and the orf1ab negative strand indicats active viral replication in the placenta.

**Conclusions:**

*Our report demonstrates active viral replication of the* SARS-CoV-2 delta-variant in the placenta associated with vertical transmission in a preterm infant.

**Supplementary Information:**

The online version contains supplementary material available at 10.1186/s12879-024-09420-y.

## Background

The B.1.617.2 (Delta) variant of SARS-CoV-2 was designated as a Variant of Concern in May 2021 by the World Health Organization. The delta-variant was noted to have an increase in transmissibility related to higher viral loads, shorter time to replication peak, and abrogated neutralization capacity compared to non-delta SARS-CoV-2 [1]. A recent study demonstrated that the delta-variant was also associated with a higher risk of severe maternal adverse outcomes than other SARS-CoV-2 variants [2]. Multinational studies showed the positivity of COVID-19 nasal swab PCR in 2–13% of neonates born to SARS-CoV-2 positive mothers [3, 4]. However, there is limited information on the specific impact of maternal infection with the SARS-CoV-2 delta variant on vertical transmission. To date, the factor(s) contributing to vertical transmission in some neonates and not others are unclear. Herein, we describe a case of a delta-variant of SARS-CoV-2 infection in a pregnant mother and her preterm newborn. The placental examination revealed SARS-CoV-2 live replicating virus in the context of vertical transmission in a preterm infant.

## Case presentation

A preterm 28 weeks and 3 days of gestational age male was born to a 42-year-old G7P4024 mother (Gravida 7, Parity- 4 full term, no preterm, two miscarriages, and four living children) with appropriate prenatal care. The mother had four previous spontaneous vaginal deliveries and no significant adverse medical history. She was transferred for management to Westchester Medical Center from an affiliated hospital due to preterm labor and flu-like symptoms for two days. She was not COVID-19 vaccinated. A nasopharyngeal swab for RT-PCR for SARS-CoV-2 was positive on admission, and the genomic sequencing revealed a delta variant of the virus. A male infant was born via classical cesarean section due to a non-reassuring fetal heart tracing. The membranes were ruptured at delivery with clear amniotic fluid. The Apgar scores were 7 and 7 at 1 and 5 min, respectively. The newborn was appropriate for gestational age; the weight was 1080 g (40th percentile), length 35.5 cm (26th percentile), and head circumference 26.5 cm (62 percentile). The placenta was collected for histopathological examination immediately after birth. The infant was admitted to the NICU and was placed in isolation due to maternal SARS-CoV-2 positivity. The neonatal nasopharyngeal swabs for RT-PCR were positive on days 1, 2, 13, 20, and 28 and became negative on the day of life 37. A viral panel including Influenza A + B, RSV, Adenovirus, Bordatella, Parainfluenza, and Rhinovirus was negative. The neonate developed respiratory distress syndrome after birth. His respiratory condition was similar to that of preterm infants of his age. He was intubated for surfactant administration for a short period and placed on non-invasive mechanical ventilation. On day 15 of life, he weaned to continuous positive airway pressure ventilation (CPAP) mode. His respiratory condition improved gradually, and he was placed in room air on day 45 of life. The baby had low white blood cells and platelet count for a week after birth. A three-day course of granulocyte-colony stimulating factor administration normalized the leukocyte count. The leukopenia and thrombocytopenia were attributed to SARS-CoV-2-associated viral illness. He got antibiotics for two days. No temperature instability, altered activity, nasal congestion, or worsening respiratory condition occurred. The rest of the neonatal clinical course was typical of other preterm newborn, including apnea of prematurity, jaundice, and feeding problems. His neurological examination and head ultrasounds were normal. There was no retinopathy of prematurity. He was discharged home at 35 weeks of post-gestational age in stable condition.

## Laboratory methods

The placenta was sent for full pathologic examination and examined grossly. At least 4 biopsies of the placenta were sampled. Placental samples were fixed in formalin, processed, embedded, and stained with hematoxylin and eosin (H&E) for histopathologic examination.

### Direct quantification of SARS-CoV-2 RNA in formalin-fixed paraffin embedded (FFPE) placental samples by ddPCR

SARS-CoV-2 gene N1 and subgenomic RNA of gene E were assayed by triplex ddPCR using One-step RT-ddPCR advanced Kit for Probes (cat# 1,664,021, BioRad, Hercules, CA) following the manufacturer’s instructions. Total RNA was isolated from 40 μm of FFPE samples (scrolls) by miRNeasy FFPE kit (Qiagen, Germantown, MD) according to the manufacturer’s instructions. N1 PCR assay (2019-nCoV RUO Kit) was obtained from Integrated DNA Technologies, Coralville, IA. The subgenomic RNA of gene E (sgE) assay was described by Bruce et al. (10.15252/emmm.202115290) and was synthesized by Integrated DNA Technologies (Coralville, IA). The sgE assay targets the sgE mRNA using a forward primer in the leader sequence of the genome, a reverse primer near the 5′ end of the E gene, and a probe that binds to the TRS junction between the leader and E gene in the sgRNA. Endogenouse gene CYC1 assay was purchased from Thermo Fisher Scientific (cat# 4448489). Three PCR assays (N1-FAM, sgE-Cy5, and CYC1-VIC) were combined with Supermix, reverse transcriptase, DTT, and RNA to a 20 µL reaction. RT-PCR amplification was carried out on a T100 Touch thermal cycler (Bio-Rad, USA) using a thermal profile beginning with reverse transcription at 46 °C for 60 min, followed by Taq polymerase activation at 95 °C for 10 min; amplification for 40 cycles of 95 °C for 30 s and 59 °C for 60 s; and concluding with 98 °C for 10 min. After PCR, the plate was analyzed on a droplet reader (Bio-Rad, Hercules, California, USA). Values for the copies/µL of target molecules were derived by QX manager Software (Bio-Rad, Hercules, California, USA). Viral load was estimated by copy number normalized by the RNA input in the RT-ddPCR reaction.

### Detection of SARS-CoV-2 RNA in Placenta FFPE samples by RNA in-situ hybridization

SARS-CoV-2 RNA in paraffin-embedded placenta tissue was detected by RNAscope® 2.5 HD Reagent Kit-RED (Cat No: 322,350, Advanced Cell Diagnostics) according to the manufacturer’s protocol. The positive strand of the SARS-CoV-2 S gene encoding the spike protein was detected with RNAscope® Probe - V-nCoV2019-S (cat# 848,561, V-nCoV2019-S, Advanced Cell Diagnostics). The negative strand of nCoV2019-orf1ab was detected with RNAscope® Probe- V-nCoV2019-orf1ab-sense (cat# 859,151).

## Results

### Placental pathology findings

The placental weight was 186 g (< 5th percentile). Placental examination revealed characteristic gross and microscopic findings of SARS-CoV-2 placental infection [5, 6] including histiocytic intervillositis, increased perivillous fibrin deposition, and villous trophoblastic necrosis.

### Detection of live SARS-CoV-2 in placental tissue

**Fig. 1:**
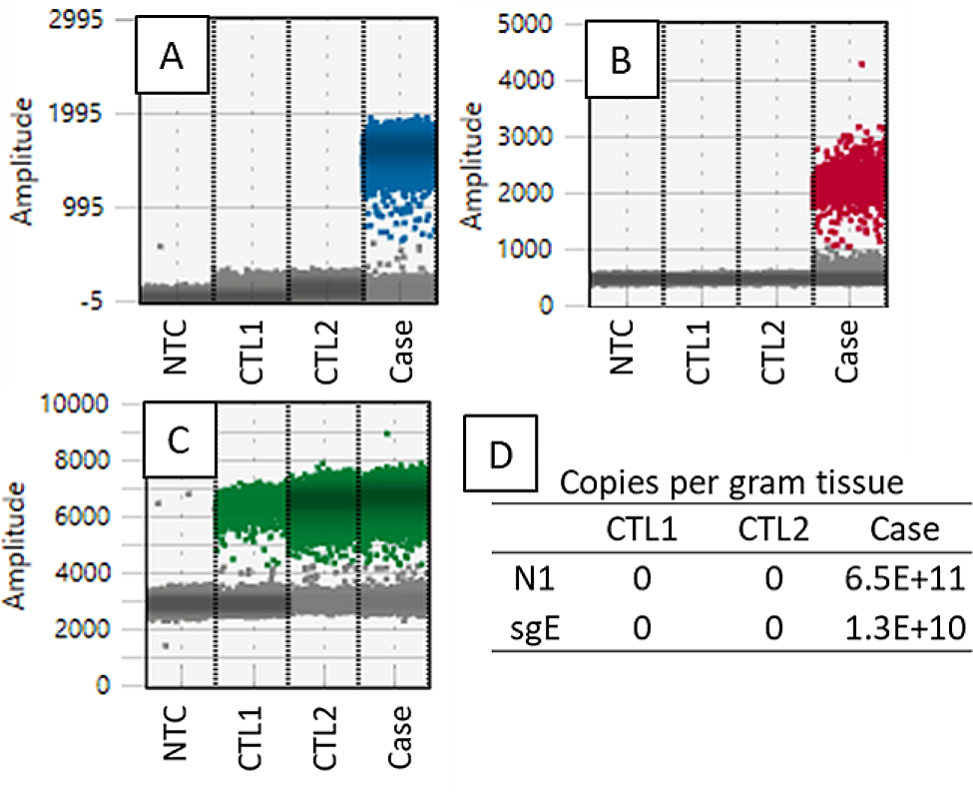
Detection of genomic and subgenomic SARS-CoV-2 in the infected placenta. One-dimensional scatterplots of positive droplets depicting the genomic RNA gene N1 (blue, panel **A**) and subgenomic gene E, sgE (red, panel **B**) indicate the presence of replicating SARS-CoV-2. The expression of CYC1 was used to the assay quality of RNA (green, panel **C**). Panel **D**. Quantitative SARS-CoV-2 viral load present as genomic and subgenomic RNA copy number per gram of placental tissue. NTC: no template control, CTL1 and CTL2: non-COVID-19 placentas collected before the year 2019. Case: infected placenta in this report

**Fig. 2:**
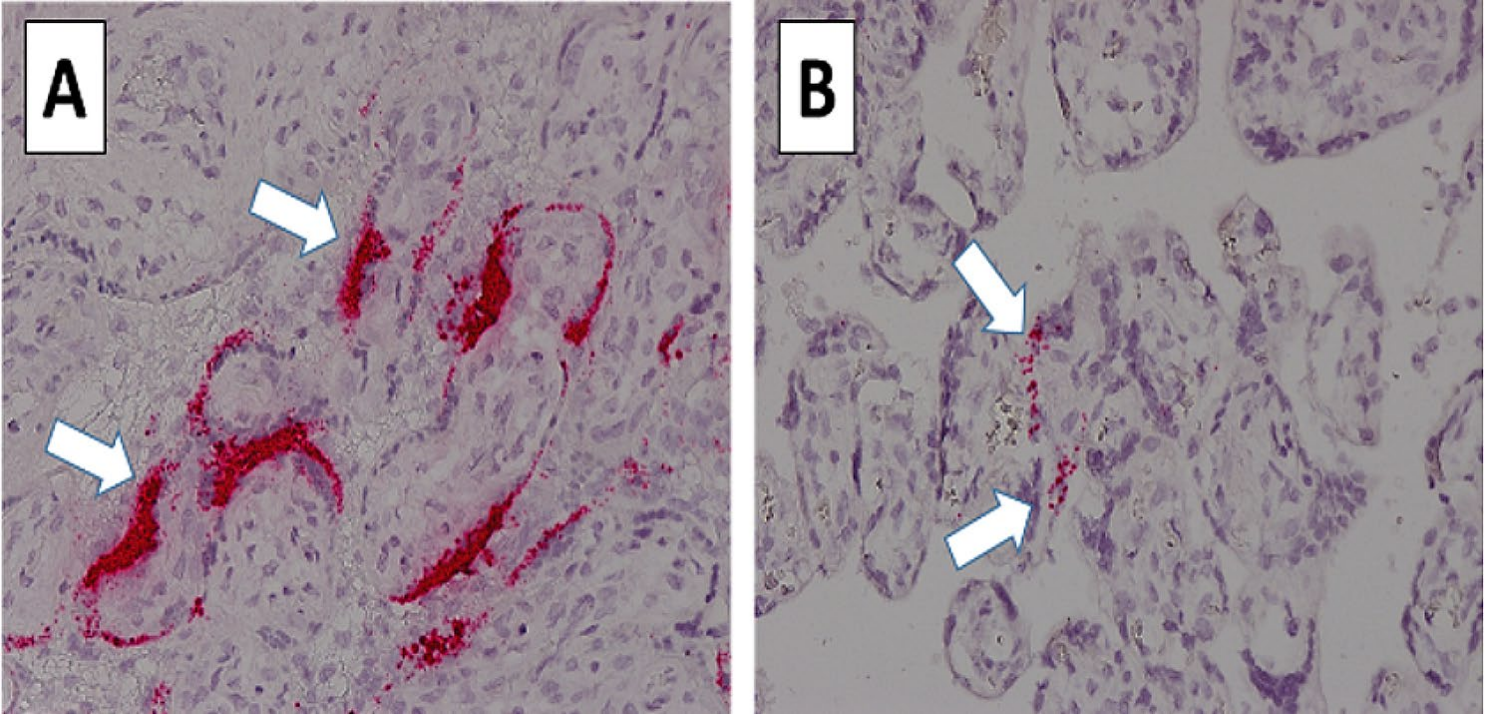
(**A**) Strong signal for the positive strand staining of SARS-CoV-2 S gene in infected placenta by RNAscope. (**B**) Signal for the the SARS-CoV-2 orf1ab negative-strand staining indicating active viral replication

We tested four different blocks from the placental samples, and all were positive for SARS-CoV-2 using ddPCR. The viral load of SARS-CoV-2 and the subgenomic gene E (sgE) indicating viral replication, are presented in Fig. 1. Using RNAscope, we detected both the positive strand of the S gene and the SARS-CoV-2 orf1ab negative strand, indicating active viral replication (Fig. 2).

## Discussion

Our report demonstrated the vertical transmission of the delta-variant of the SARS-CoV-2 virus. The detection of the positive strand of the S gene and the negative strand of SARS-CoV-2 orf1ab indicates the presence of the virus gene and active viral replication.

Postnatal acquisition of the virus is usually implied by contact with caregivers or the sterility of the delivery room. In our case, the patient was delivered by cesarian section and handled by well-protected staff. Detection of the SARS-CoV-2 by RT-PCR in a nasopharyngeal swab of a neonate on the first day of life and the detection in the placenta confirms vertical transmission. Prolonged shedding of the virus may occur due to the slow degradation of the nucleic genome of the virus [7]. The neonate in our study tested positive for SARS-CoV-2 by nasal swab RT-PCR for four weeks after birth. The prolonged positive results are similar to the findings of Carmo et al.; and others. They exhibited positive test results in 69.8% of patients until 20 days postnatal age and 34% for 40 days [8].

Previous studies have shown inflammatory placental changes in SARS-CoV-2-positive mothers [9]. Similar findings were illustrated in our study’s histopathological examination of the placenta. Increased peri-villous fibrin deposits, intervillositis, and necrotic syncytiotrophoblastic changes indicate a placental response to maternal viremia [10]. Typically, the placenta is a substantial barrier to the entry of pathogens. Immunological components such as cytokines, neutral killer cells, placental macrophages, and CD4 lymphocytes provide an effective barrier against infections. Furthermore, the lack of gap junction in syncytiotrophoblastic cell layers produces a physical barrier to the entry of invading agents [11]. The current omicron variant has higher infectivity compared to the delta variant [12]. The published pathological reports showed similar vascular malperfusion and decidual arteriopathy findings in Delta and Omicron placental samples [13]. The Omicron variant of SARS-CoV-2 causes mild clinical symptoms and few deaths [14]. To our knowledge, the detection of the Omicron variant in the placenta has not been reported.

Our study illustrated the placental SARS-CoV-2 spike protein mRNA in a SARS-CoV-2 positive mother-neonate dyad. The identification of SARS-CoV-2 in the placenta depends on the maternal viral load [5]. In the process of SARS-CoV-2 virus replication, a nested set of subgenomic RNA (sgRNA) is generated to express the viral structural proteins. Because sgRNAs are generated only during replication, the detection of sgRNAs has been used as a marker of active viral replication [15, 16]. In this study, we employed a quantitative measure of the viral replication in placental tissues using ddPCR, a state-of-the-art technology that provides absolute quantification of target mRNA copies per input sample. In our case, not only the placental viral load was high, but the high number of sgE indicates that active viral replication might have contributed to the vertical transmission.

Evidence of SARS-CoV-2 presence in the human placenta has been observed by several labs [9, 10, 17], including ours [5, 18]. These studies detected SARS-CoV-2 positive-sense RNA, indicating the presence of the virus in the placenta. However, few reports confirmed the detection of SARS-CoV-2 negative-sense RNA, indicating rare viral replication [19–21]. SARS-CoV-2 is a positive-sense, single-stranded RNA virus, and its negative-sense template is formed during the replication stages. The RNAscope antisense probe can detect the spike protein’s positive sense of mRNA and its negative template [19]. Open reading frames (ORF1ab) sequences form several nonstructural proteins (nsp) that preserve and store genomic information for the synthesis and replication of viruses [21, 22]. In the current study, the detection of SARS-CoV-2 ORF1ab negative strand indicates SARS-Co-2 virus replication, which was not shown previously with the delta virus.

## Conclusion

This report is the first to demonstrate active viral replication of the SARS-CoV-2 delta-variant in the placenta associated with vertical transmission in a preterm infant. Viral load and the type of variant of SARS-CoV-2 may be important factors that overwhelm the placental protective mechanisms against the SARS-CoV-2 infection, leading to active viral replication and vertical transmission. Further studies are needed to evaluate the correlation of placental active viral replication to vertical transmission in neonates.

### Electronic supplementary material

Below is the link to the electronic supplementary material.

Supplementary Material 1

## Data Availability

No datasets were generated or analysed during the current study.
