# Supplementary material for: Vertical transmission of SARS-CoV-2 delta-variant in a preterm infant
Source: BMC Infect Dis. 2024 May 28;24:537. doi: 10.1186/s12879-024-09420-y (PMC11134764; doi:10.1186/s12879-024-09420-y)
Supplement: Supplementary file 1 — Supplementary Material 1 [file 12879_2024_9420_MOESM1_ESM.docx]

Addressing the consent issue:

We fully understand and adhere to your journal's editorial guidelines regarding obtaining written, signed informed consent from patients or their legal guardians for case reports before submission. In line with this, we took the necessary steps as outlined in our submission. This unique case was written over two years post-discharge of the patient. Despite numerous efforts, we were unable to contact the patient's family. Additionally, the pediatrician involved mentioned that after the initial office visit, they were also unable to contact the mother despite multiple attempts, leading to the belief that she may have relocated.

This case was presented to the New York Medical College institutional review board (IRB Protocol #19936). Upon review, the IRB concluded that our research did not fall under the category of human subjects research as per 45 CFR 46 Federal Regulations. Consequently, the IRB has granted an exemption from the requirement to obtain informed consent.

We have meticulously ensured that all potentially identifying information about the infant is omitted from the case report. This includes names, photographs, or any personal details that could lead to identification. If you identify any content in the publication that might still inadvertently reveal the subject's identity, please inform us and we will remove it promptly.

Considering these circumstances, I kindly request that this matter be escalated to the journal's editor for further evaluation, affirming our compliance with the stated editorial policies.
